# Place identity in a personality psychological context: place identity orientation and its validating associations with nature connectedness and pro-environmental behavior

**DOI:** 10.3389/fpsyg.2024.1424381

**Published:** 2024-12-19

**Authors:** Iván Zsolt Berze, Andrea Dúll

**Affiliations:** ^1^Doctoral School of Psychology, ELTE Eötvös Loránd University, Budapest, Hungary; ^2^Institute of People–Environment Transaction, ELTE Eötvös Loránd University, Budapest, Hungary; ^3^Department of Sociology and Communication, Budapest University of Technology and Economics, Budapest, Hungary

**Keywords:** place identity, identity orientations, place identity orientation, nature connectedness, pro-environmental behavior

## Abstract

In the frame of the inner–outer (personal–social) dichotomy in theories of social or personality psychology, it is argued in this study that, as a new approach, it is reasonable to examine individual differences in tending to orient toward another outer facet, not just social, i.e., the surrounding socio-physical environment while defining the self-concept—first performing it at the level of place-scaled meaningful settings, thus, interpreting place identity in the context of identity orientations. The present research aimed to theoretically and methodologically support a new concept based on this approach: place identity orientation (PIO). For the latter purpose, the development process of the questionnaire measuring it, which was based on supplementing the Aspects of Identity Questionnaire (AIQ-IV), was completed. In addition, the new construct was validated based on the relationships discussed in literature between place identity, connectedness with a larger-scaled environment, i.e., nature, and pro-environmental behavior (PEB), moreover, identity orientations and behavior. In our first scanning step of validating PIO, a partial mediation between PIO and PEB through nature connectedness was found among 1,281 adults. It suggests that the relative importance of the relationship with places in self-definition has a distinct role in addition to nature connectedness in behaving pro-environmentally. Limitations and future research directions (e.g., using PIO to examine people–environment fit in several settings) are also discussed.

## 1 Introduction

### 1.1 Place identity

Environmental psychology (Bechtel and Churchman, 2002; Donald, 2022; Proshansky et al., 1970; Steg and de Groot, 2018; Stokols and Altman, 1987), in addition to the role of the social environment, also emphasizes the importance of the physical (built, natural, and contemporary virtual) environment in the psychological processes of the person. As the physical environment is also the scene of social relationships that determine a person’s behavior and thinking about themselves, the impact of the physical environment on the person can hardly be separated from the influence of their social milieu. In addition, each physical environment is also socially determined: environmental psychology defines the environment as socio-physical (Stokols and Shumaker, 1981). The physical environment is not merely a passive and static background but a, partly precisely due to its user, constantly changing context that has an effect on its user in every case, even if this effect is often non-conscious. As a result of the transactional (Werner et al., 2002) relationship with the environment and at the same time as a precondition of it, the person’s mental representation of this relationship necessarily develops that may vary in strength and quality, but in each case is complex and dynamic. Although people are always related to their environment, not all elements of the physical environment become important to them: certain environmental settings (spaces) are endowed with meanings, and the spaces thus become places (Canter, 1977).

One of the most important constructs of environmental psychology, place identity (Proshansky, 1978; Proshansky et al., 1983), develops based on the transactional—on people’s side: perceptual and cognitive—processes between people and their environment and describes the phenomenon when places important to a person become related to their self-identity. Place identity can be described as those dimensions of self-identity that are in “relation to the physical environment by means of a complex pattern of conscious and unconscious ideas, beliefs, preferences, feelings, values, goals, and behavioral tendencies and skills relevant to this environment” (Proshansky, 1978, p. 155), i.e., as a pattern of cognitions (memories, concepts, interpretations, and ideas) and associated feelings about specific past, present, and even imagined physical environments important to the person. Proshansky et al. (1983, p. 63) discussed place identity’s “role in shaping the behavior and experience of the person in given physical settings,” even if actual behavior occurs depending on a host of other factors, not only the necessary place-related cognitions. Regarding the terms of Paasi (2009), who suggested differentiating the place identity of people and a place, defining the latter as a pattern of those features of a place that people use to distinguish it from other places, our research focuses on people’s place identity.

Although Proshansky, by mentioning “role-related identities” (Proshansky, 1978, p. 155), linked place identity to social identity, he did not clarify their exact relationship, and after decades of investigating place identity, there still is no clear consensus about their connection (c.f., Bernardo and Palma-Oliveira, 2013; Hinds and Sparks, 2008; Knez and Eliasson, 2017). Similarly, no consensus can be found regarding the connection of place identity with other concepts discussing the relationship between person and environment (e.g., place attachment, place dependence, or sense of place; Devine-Wright and Clayton, 2010; Dúll, 2017; Manzo, 2003; Peng et al., 2020; Rollero and De Piccoli, 2010). The issues of the relationship between place identity and place attachment are relevant to the context of our research. “Place attachment refers to bonds that people develop with places” (Lewicka, 2008) and is “usually understood as emotional ties” (Lewicka, 2010). Indeed, at least partially, due to differences in the interpretation of the nature of this bond (emotional or general), examples can be found in literature arguing that (1) the two concepts are the same construct (e.g., Brown and Werner, 1985); (2a) place attachment is a part or core of place identity (e.g., Belanche et al., 2017; Droseltis and Vignoles, 2010; Korpela, 1989; Lalli, 1992; Strandberg and Styvén, 2024); (2b) place attachment is an antecedent to place identity (e.g., Hernández et al., 2007; Knez, 2014); (3) place identity is part/dimension of place attachment (e.g., Boley et al., 2021; Kyle et al., 2005; Maricchiolo et al., 2021; Ramkissoon et al., 2013a: place attachment as a higher-order concept, including place affect measured similarly as place attachment by other research; White et al., 2008; Williams and Roggenbuck, 1989), and (4) both place identity and place attachment are parts/dimensions of a higher-order concept (e.g., sense of place: Hummon, 1992; Jorgensen and Stedman, 2001).

As a new approach nested in the intersection between two fields of psychology, we argue that place identity as an environmental psychological construct can also be interpreted in a personality psychological context, i.e., as an individual tendency to orient toward the place-scaled socio-physical environment(al settings) when constructing self-definition, thus establishing a specific place-independent concept: the place identity orientation (detailed later). Based also on Proshansky’s original definition, our questionnaire, which aims to measure and contribute to validating this new concept, includes items that refer to the importance of emotional connection with places in self-definition. This also meets the conceptual thinking about place attachment, which considers it emotional ties and part of or antecedent to place identity. However, our research is not committed to verifying the validity of our thinking on the relationship between place identity and place attachment.

### 1.2 Place identity and nature—identity connection

Another main issue is the comparison of place identity and environmental identity. As Devine-Wright and Clayton (2010) discuss, “place” and “environment” (in this context, “nature”) vary in terms of specificity and scale/boundaries: the former refers to a specific location, and the latter is more general, referring to natural ecosystems. Similarly, place identity and environmental identity also differ in their generalness and their related geographical scope and boundaries. In addition, they also vary by the extent of the specification and localization of the involved experiences and memories and possibly by other differences in cognitive structure (Devine-Wright and Clayton, 2010).

Several concepts exploring the connection between nature and person can be found in literature (Tam, 2013; Whitburn et al., 2020). As Whitburn et al. (2020) pointed out, the concept of connection to nature is essentially related to self-identity, which is expanded by including nature and experiences of belonging to it. Even if, as might be complemented, its definitions or some measuring items do not explicitly emphasize this identity-related feature. For example, Brügger et al. (2011) consider it an attitude, largely avoiding self-reflection. Some concepts of connection to nature are multi-faceted: in addition to the cognitive aspect, they incorporate affective, spiritual, behavioral, experiential, and/or attitudinal facets of the connection with nature. Among others, Nisbet et al.’s (2009) nature relatedness and Clayton’s (2003) environmental identity can be classified between them. The latter “is one part of the way in which people form their self-concept: a sense of connection to some part of the non-human natural environment” (Clayton, 2003, p. 45), also including emotions, values, attitudes, and behaviors related to nature. Regarding Clayton’s definition, we might argue that environmental identity can be paralleled with Proshansky’s original definition of place identity: environmental identity can be simultaneously considered both a specific (as it applies to one of the many outer environments of the person) and a non-specific (as it focuses on nature in general, instead of specific natural settings) variety of Proshansky’s place identity term.

Other concepts of nature–identity connection focus on one facet of the connection, for example, emotions [e.g., connectedness to nature (CTN), Mayer and Frantz, 2004] or the cognitive aspect [e.g., inclusion of nature in self (INS), Schultz, 2001, 2002]. Mackay and Schmitt (2019) pointed out that both CTN and INS are adapted from close relationships and intergroup relations. Schultz’s (2002) connectedness with nature “refers to the extent to which an individual includes nature within his/her cognitive representation of self” (Schultz, 2002, p. 67), thus focusing on a sense of oneness with nature. The concept of Schultz can be considered parallel to one of the four place identity dimensions reported by Droseltis and Vignoles (2010), i.e., self-extension. Due to its feature emphasizing the cognition of the connection and its shortness, this scale was applied to measure the relationship between identity and nature in this research.

### 1.3 Associations between environmental-relevant identities and pro-environmental behavior

Promoting pro-environmental behavior (hereinafter: PEB), defined as “behavior that harms the environment as little as possible, or even benefits the environment” (Steg and Vlek, 2009, p. 309) is crucial nowadays in achieving the aim of reducing human activities’ harmful impacts on nature. PEB (or intentions for it) may be classified into more interrelated types, considering, for example, the purposes mentioned in the definition (behavior to benefit nature or acts to avoid harming it: c.f., Lange and Dewitte, 2019), the behaving actors (e.g., individual, collective, and policy support behavior: Walker et al., 2015; green purchase, good citizenship behavior, and environmental activist behavior: Lee et al., 2014) or the environment/setting (place-specific or general: Halpenny, 2010). The PEB measurement methods are also an important issue in the context of our study (self-report measures, field observations, or laboratory observations: Lange and Dewitte, 2019). In our study, we self-reportedly measured PEB as a general, individual behavior focused on avoiding harming the environment, which is considered at the scale of the global ecosystem.

In Udall et al.’s (2020) theoretical framework, place-focused identity means one of the three levels of identity relating to PEB (in addition to the individually focused and group-focused identity). They stressed the relationship between identity and behavior, existing primarily by their shared meaning. Discussing the relationship between environmental-relevant identities and PEB, the issue of the specificity of the behavior is pivotal. As Devine-Wright and Clayton (2010) noted, very specific identities may also be important within a narrow range of contexts. Place identity has a role both in the regulation of behavior and the processes of people–environment fit (Proshansky et al., 1983) and in the maintenance of a sense of continuity to identity (Twigger-Ross and Uzzell, 1996)—in place-scaled settings where usually PEB can be executed in the everyday. Although several studies can be found exploring the positive association of place identity with PEB (e.g., place-specific: Faccioli et al., 2020; Swim et al., 2014; general: Halpenny, 2010; Vaske and Kobrin, 2001), the positive relationship is not justified by other researchers (e.g., Junot et al., 2018; Ramkissoon et al., 2013b). Nevertheless, the existence of a positive association between connection with nature and PEB is much less doubtful (e.g., Alcock et al., 2020; Mackay and Schmitt, 2019; Martin et al., 2020; Obery and Bangert, 2017; Sierra-Barón et al., 2023). In the frame of a meta-analysis, Whitburn et al. (2020) conducted a meta-analysis to thoroughly review the results of 26 studies published on this topic up to 2018.

### 1.4 Identity orientations

Identity orientations can be interpreted as the relative importance or value of various identity attributes for individuals when they construct their self-definitions (Cheek, 1989). The theory of identity orientations is rooted in the inner–outer metaphor (personal–social dichotomy) found in social or personality psychology theories and conceptions of identity’s content components. We can mention, for example, the spiritual and social self (James, 1890), the private and public self (Greenwald and Pratkanis, 1984), the private and interpersonal self (Neisser, 1988), public and private self-consciousness (Fenigstein et al., 1975), individual, relational, and collective self (Sedikides and Brewer, 2001), the theoretical model of multiple dimensions of identity (Jones and McEwen, 2000), and so on.

Miller (1963) distinguished personal and social identities, containing views of the self, a sense of continuity and uniqueness, and social roles and relationships, respectively. Based on Miller’s categories and considering them as two endpoints of the same theoretical dimension, Sampson (1978) argued that, when defining their self-concept, individuals tend to orient toward either the internal (intrinsic) aspects of themselves or the external (social) environment. Stressing that identity doubtlessly incorporates both personal and social aspects for most individuals and that there are individual differences in their relative importance, Cheek and Briggs (1982), based on the result of the correlational analysis of the two subscales of Sampson’s questionnaire in the frame of the development process of the Personal and Social Identity Scales (AIQ, Cheek and Briggs, 1982), suggested that the two orientations should be considered as representing two independent dimensions. During several steps of improving their questionnaire, Cheek et al. developed the fourth version (AIQ-IV, Cheek et al., 2002) of the Aspects of Identity Questionnaire, which measures four identity orientations: personal, social, collective, and relational—a more recent study by Cheek and Cheek (2018) reported that to avoid confusion based on the use of social identity to refer to group membership in social identity theory, they renamed social to public identity orientation. Thus, the AIQ-IV measures individual differences in the subjective importance and value of the following in self-definition: (1) personal values, thoughts, feelings, goals, views of self, sense of continuity and uniqueness; (2) public aspects, perceptions of how he/she is perceived or thought about by others, (3) (involuntary social) group membership (family, ethnicity, religion, nation, and mother tongue), and (4) close relationships (friendship and partnership) and the person’s role in them, respectively. In the context of the research with the questionnaire, highlighting that AIQ-IV measures subjective importance, rather than subjective evaluation or liking, of different aspects of identity, Cheek and Cheek (2018) evolved a four-dimensional model of identity/self-concept that incorporates two parts: the independent self (representing the personal self) and the interdependent self (consisting of the relational, public, and collective self). As Fenigstein et al. (1975) described personal and social self-consciousness as dispositions and the concepts of personal and social identity orientation (and their measurement tool) were developed in the context of private and public self-consciousness theory (Cheek and Cheek, 2018), identity orientations can, in our view, also be considered to have disposition feature. As Cheek and Cheek (2018) stressed, several research found a relationship between identity orientations and behavior (e.g., public/personal identity orientation and social behavior: Leary et al., 1986; collective identity orientation and social behavior Ryder et al., 2000; public identity orientation and health behavior: Leary and Jones, 1993; Hagger et al., 2007).

### 1.5 Place identity orientation

Environmental psychology stresses the role of the physical environment, in addition to the social one, in people’s everyday functioning. Self-definition can also undoubtedly be shaped and enriched by several-scaled physical environments: on the larger scale, nature, and on the smaller scale, the socio-physical places surrounding the person. Nonetheless, it can be argued that the “external” side of the inner–outer dichotomy mentioned above lacks the physical environment, with very few exceptions with different foci than ours (e.g., Little, 1972). After interpreting the relative importance of social aspects of identity, it seems reasonable to investigate individual differences in tending to orient toward the surrounding physical environment, firstly interpreted at the level of the geographical scope/boundaries and meaningness of places while defining the self-concept (for another interpretation level and its methodological difficulties, see Future research directions below). It represents a new approach in both personality and environmental psychology: in personality psychology, it expands the outer side of the inner–outer dichotomy, whereas in environmental psychology, it extends and interprets the concept of place identity to the level of a more general tendency. Previous research has mainly studied place identity related to specific or favorite places. It was examined, for example, related to cities of different sizes (e.g., Casakin et al., 2015), regions (e.g., Belanche et al., 2021; Carrus et al., 2005), nested/interconnected places (e.g., Bernardo and Palma-Oliveira, 2013; Hernández et al., 2007), specific natural settings (Kyle et al., 2005; White et al., 2008), school settings (e.g., Marcouyeux and Fleury-Bahi, 2011), home (e.g., Chow and Healey, 2008), and workplace (e.g., Elsbach, 2003; Rooney et al., 2010). Examining the individual differences in the relative importance of the relationship with places in self-definition, i.e., investigating the tendency to orient toward places when constructing self-definition, requires measuring place identity “in general,” i.e., without referring to particular places. In this sense, place identity orientation can be interpreted as a generalized term for specific place identities, and the construct is simultaneously considered the partial basis for developing them. To interpret place identity in a personality psychological framework, i.e., in the context of identity orientations, the AIQ-IV was applied as a frame: during the first two steps of our research series (Berze and Dúll, 2018, 2021), the items of AIQ-IV translated into Hungarian were supplemented with items aiming to measure place identity orientation (hereinafter: PIO) and the dimensionality and psychometric features of the different (improved) versions of the new questionnaire were examined.

The issues discussed above in justifying the relationship between place identity and PEB might arise from the different scales and specificity of the two constructs. Interpreting place identity on the level of a more general tendency, i.e., investigating place identity orientation, might allow examining associations of place identity with a general (not place-specific) PEB in line with environmental identity, which is also general but in a different sense than place identity orientation: regarding its “targeted” physical environment, nature in general (instead of specific natural places). By studying the relationship between the “general” place identity orientation and the general PEB, which refers to protecting nature but is executed in everyday environments, i.e., places, it is inevitable to encompass the closely related “general” concept, the environmental identity concerning nature, in the investigation. By executing a mediation analysis, we can reveal whether the association, if it exists, between the general tendency to orient toward (both natural and built environmental) places when constructing self-definition and pro-environmental behavior is in itself or just through environmental identity. This mediated relationship, in addition, can validate our new concept.

### 1.6 Hypotheses

In the present research, we aimed to argue the necessity of the inclusion of the physical environment in the inner–outer metaphor in identity context and validate our new construct, place identity orientation. The validation was based on using a valid questionnaire measuring it and on the relationships discussed above between place identity and PEB and between identity orientations and behavior. The former aim means, consequently, also adding the physical environment to Cheek and Cheek’s (2018) model. However, our research was committed to supporting our new concept theoretically and methodologically, rather than building and testing a new model. Regarding the latter purpose, in addition to the arguments in H4 below, we decided to validate our concept with helping of PEB because a questionnaire that measures “general place-scaled environmental behavior” discussed in line with the functions of place identity by Proshansky et al. (1983) is not available yet. Nevertheless, this behavior would provide a stronger validating factor. The following hypotheses have been formulated according to our aims.

H1: We expected that, as a result of the content modifications aiming to eliminate error covariances among PIO items and wording refinements of some translated original items of AIQ-IV in this research step (see later), a valid and reliable Hungarian questionnaire was developed for measuring place identity orientation and the four original identity orientations.

H2: Considering both the conceptual similarity and the scope/boundary-based differences between place identity and environmental identity, PIO was hypothesized to be positively associated with nature connectedness, i.e., the extent of the inclusion of nature in self.

H3: Regarding the behavior-regulating feature of place identity, we hypothesized that PIO is positively associated with pro-environmental behavior (PEB).

H4: Based on the premise that effective PEB requires effective and adequate dealing with place-scaled environmental settings generally (not only with nature but several settings of the everyday built environment of the person), and according to Proshansky et al. (1983), this dealing can be supported by place identity, it was also expected that PIO is associated with PEB partially directly and not only mediated by nature connectedness. (The positive association between nature connectedness and PEB was confirmed in several research.)

Since the aim of including the other four identity orientations in the analyses was exploring the independence of results in PIO-PEB associations from them, no hypotheses on their relationship with PEB were formulated.

## 2 Method

### 2.1 Sample

The completion of the online questionnaire pack lasted approximately 25 min, and was anonymous. The participants could start it only after reading the research ethics information and consenting to participation. Data collection was carried out in the consecutive semesters of 2022 and 2023 in the framework of two university course units, which offered students the opportunity to earn credit by participating in several research projects. In one of them, students were asked to recruit participants with the required characteristics, in this case, over the age of 25. Due to recruitment requirements, the sample was composed of not only university students. More than one completion by the same person was prevented.

The completion of the questionnaire pack occurred by 1,674 participants. After deleting non-consenting persons’ data rows and blank/severely incomplete data rows, a database containing the answers of 1,597 persons (65.7% women, age: M = 30.98, SD = 13.913, ranged between 18 and 86 years) was created to analyze the data, reaching thus confidently an adequate sample size (c.f., MacCallum et al., 1999) to execute the factor analyses of the 49 items of the questionnaire measuring identity orientations. Although some participants did not answer the items measuring pro-environmental behavior and nature connectedness, the path analysis, including the answers to these items, was able to be executed on an adequately large and valid subsample (*N* = 1,281).

### 2.2 Measurement tool

The parts of the more-than-100-item questionnaire pack included in the present analyses were the following.

#### 2.2.1 Place identity orientation and other identity orientations

These constructs were measured by a questionnaire developed and modified in and after the two previous steps of our research series. The base of this questionnaire was the translated fourth version of the Aspects of Identity Questionnaire (AIQ-IV, Cheek et al., 2002) which was supplemented with our place identity orientation (PIO) items.

As the development process conducted in the first (Berze and Dúll, 2018) and the second (Berze and Dúll, 2021) steps of our research series is published in Hungarian, and only an abstract is available about the English-language conference presentation (Berze and Dúll, 2022) summarizing it, a brief review of the development process is presented in the following.

In the first step of our research series (Berze and Dúll, 2018), nine new PIO items were formulated and inserted into the Hungarian-translated version of AIQ-IV. In our developmental work, we had to keep crucial methodological considerations in mind:

- the designation of the places: as the aim was the interpreting of place identity in a personality psychological framework, i.e., assessing the individual differences in the relative importance of the relationship with places in the self-definition (in other words, measuring a tendency to orient toward places generally when constructing self-definition), particular places (e.g., home and office) have not been mentioned; we used the general word “place,” emphasizing in some items that both built and natural environment can be encompassed, and, by using an expression (“egy-egy,” almost corresponding in English to “some”) that more than one but not more than a couple of places can be considered,
- the content of the items: aspects/dimensions of place identity, encompassed in place identity questions/items in research (e.g., feeling the place is part of the person, identifying with the place) and mentioned in literature (e.g., memories, meanings, and emotional bonding), were intended to be included that can be easily interpreted without referring to a particular place—thus, e.g., aims and behaviors were excluded,
- the formulation was conducted in the style of the AIQ-IV items,
- the method of insertion: the new items were inserted in a scattered manner.

The resulting questionnaire was tested in three versions in samples of university students. The second version contained reformulations according to the results of the exploratory factor analysis (EFA) of first version, whereas the third version, containing only the translated item of the original AIQ-IV, justified that the insertion of PIO items did not compromise the integrity of the original questionnaire.

In the second step of the research series (Berze and Dúll, 2021), modifications to the questionnaire were implemented:

- considering the first place of the factor of PIO items in the factor structure of EFA and the error covariances between some PIO items showed in the confirmatory factor analysis (CFA) in the previous step, more differentiated but still general designations were used instead of “place,” e.g., “physical environment”; in addition, other aspects of place identity were included in the items, e.g., self-extension (Droseltis and Vignoles, 2010),
- some reformulations were performed in the case of the original questionnaire’s items that showed uncertainties in the factor structure to also improve the fit to the factor structure of the original questionnaire—especially by the collective identity orientation items, pondering the differences in the sociocultural context between the USA and Hungary,
- considering the low explained variance (below 40%) in the EFA in the previous step, some of the out-of-scale items of the original questionnaire were deleted, and the PIO items were rearranged for better distribution.

To increase the validity of the modified questionnaire, it was completed by two adult samples, instead of university students, and the formulation was refined more between the two versions.

According to the results of the EFA and CFA, the modifications remedied some issues above, resulting in second place for the factor of PIO items in the factor structure of EFA and higher explained variance (43.494%).

Although the modifications remedied some issues in the second research step mentioned above, further alterations seemed reasonable (e.g., although the results of CFA showed a better fit to the original questionnaire’s factor structure than in the first version, it needed more improvement; in addition, error covariances between PIO items still remained). Therefore, the following modifications were conducted in the recent research step.

- Due to the remaining error covariances, content adjustments were executed in cases of some PIO items. As a result, the final 9 PIO items covered the following place identity aspects/dimensions emphasizing mainly the cognitive level of the concept: general attachment, identification with places, self-extension (feeling that a place is a part of the self), memories, emotional bonding, meaningfulness, place-self congruity, environmental fit (feeling of being a part of a place), and environmental preference. As an example of the PIO items, the respondents were asked to consider how important the following is to their sense of who they are: “My attachment to some natural or built environmental places.”
- Due to the uncertainties in the factor structure (e.g., loading in an inappropriate factor for the content, low factor loadings), wording refinements were performed in some translated items belonging to the original questionnaire, e.g., in item 10 (public identity orientation) or item 27 (collective identity orientation).

The questionnaire contained 49 items answerable on a 5-point Likert scale (ranging from “not important” to “strongly important,” later coded as 1 to 5). Higher scores on the subscales indicate a greater extent to which the particular identity aspect is important in the sense of who the person is.

#### 2.2.2 Pro-environmental behavior

Self-reported general, thus, not place-specific PEB was measured via six items adapted from PEB items used in another research project in our Lab. These latter items were created based on measurement considerations (c.f., Mónus, 2021) and inspired by items from several questionnaires assessing pro-environmental behavior. Our items could be answered on a 6-point Likert scale (ranging from “strongly not typical for me” to “strongly typical for me,” later coded as 1 to 6). The content of the items covered separate waste collection, saving water and energy, preference for environment-friendly products and mobility, use of things as long as they are useable, and avoiding the usage of single-use plastic products. A higher score on the scale (unidimensionality was confirmed; see later) indicates that environment-friendly behavior is more typical for the person.

#### 2.2.3 Nature connectedness

To measure this construct, the Inclusion of Nature in Self Scale (INS, Schultz, 2002) was applied. It consists of seven Self–Nature circle pairs overlapped to varying degrees ranging from separate (coded as 1) to completely overlapping (coded as 7). The Hungarian version of INS was developed during another research project of our research group. A higher value indicates a greater extent of inclusion of nature in self.

### 2.3 Data analysis

After creating the whole sample by pooling the samples of the two university courses and then randomly halving it, exploratory factor analysis (EFA) with principal axis factoring extraction method and Promax rotation was conducted on the 49 identity orientation items using the responses of the first random half (*N *= 798) of the whole sample (with pairwise deletion of cases with missing values) to reveal the factor structure of our modified questionnaire. Subsequently, confirmatory factor analyses (CFA) based on structural equation modeling (SEM), using MLR estimates, were performed, encompassing the answers of the second random half (*N* = 799) of the whole sample on the 49 items to examine the fit of data to the factor structure of the original questionnaire and to the factor structure resulting from the EFA performed in the sample’s first random half. Only factor loadings above 0.35 (c.f., Hair et al., 2010, p. 115) were interpreted.

Regarding the fit indices, a value of RMSEA (root mean square error of approximation) below 0.05 indicates an excellent fit, and a value between 0.05 and 0.08 shows a good fit. The significant deviation of the RMSEA from 0.05 is indicated by the value of Cfit: not significant (*p* > 0.05) probability values indicate acceptable model fit (Browne and Cudek, 1993). For CFI (comparative fit index) and TLI (Tucker–Lewis index), a value above 0.9 is expected (Brown, 2006), while in the case of SRMR (standardized root mean square residual), a value below 0.08 indicates a good fit (Kline, 2005).

Principal component analysis (PCA) with Promax rotation was executed using the answers on the PEB items to confirm that calculating a summed score for them is allowed.

Reliability analyses were executed in the case of all (sub)scales.

Finally, a saturated path model with observed variables was used to reveal the associations of the place identity orientation with INS and PEB. To explore whether these associations are independent of the four other identity orientations, age, and gender, an adjusted saturated model including these variables as predictors was also examined. Although more research considering and using the INS score as a continuous variable can be found in literature (e.g., McConnell and Jacobs, 2020; Schultz and Tabanico, 2007), due to the number below 11 (c.f., Nunnally and Bernstein, 1994) of the INS scores, the mediator variable (i.e., INS score) was considered categorical (ordinal) in our research. Thus, in the path models, we conducted probit regression with WLSMV robust estimation and delta parameterization. We interpreted the standardized regression coefficients (βs) and the total, direct, and indirect effects based on the STDYX output.

SPSS version 28.0 (IBM Corp, 2021) was used for EFA and PCA, and Mplus version 8 (Muthén and Muthén, 1998–2017) was applied for CFAs and path models.

## 3 Results

The EFA of identity orientation items (Kaiser–Meyer–Olkin measure of sampling adequacy: KMO = 0.920, Bartlett test: *p* < 0.001) in the first half of the sample resulted in five factors that had an Eigenvalue higher than 1 and were interpretable according to the Scree Plot, explaining 43.753% of the variance (the 6^th^ factor contained only two out-of-scale items). The factor structure interpreting the factor loadings only above 0.35 is presented in Table 1. The results show that (1) the PIO items loaded into a single factor, which had the second place in the structure, (2) the 1^st^, 3^rd^, and 4^th^, i.e., the relational, public, and personal identity orientation factors contained all the adequate original items—the exception is the 5^th^, i.e., the collective identity orientation factor which two of the original collective items missed from, (3) there were no items having cross-loadings, and (4) the variance explained is slightly higher than in the previous research step, however, only five factors were interpreted in the present step.

**Table 1:** Results of the exploratory factor analysis (EFA) of identity orientation items which was conducted on the data of the first random half (*N* = 798) of the whole sample.

Items^a^	Factors				
	F1	F2	F3	F4	F5
R39	0.801				
R45	0.781				
R47	0.765				
R41	0.709				
R38	0.708				
R43	0.672				
R35	0.627				
R29	0.580				
R32	0.579				
R24	0.533				
PL30		0.824			
PL21		0.796			
PL34		0.778			
PL25		0.740			
PL48		0.686			
PL7		0.637			
PL44		0.613			
PL40		0.583			
PL17		0.570			
PE15			0.766		
PE31			0.735		
PE36			0.656		
PE2			0.645		
PE23			0.635		
PE28			0.633		
PE12			0.627		
PE9			0.605		
PE5			0.483		
PE20			0.450		
PU13				0.849	
PU16				0.747	
PU6				0.704	
PU19				0.696	
PU3				0.632	
PU22				0.395	
PU10				0.392	
C33					0.858
C46					0.642
C8					0.597
C11					0.492
C27					0.452
C4					0.382
Correlation coefficients between factors					
	F1	F2	F3	F4	F5
F2	0.294				
F3	0.570	0.143			
F4	0.341	0.295	0.242		
F5	0.212	0.441	0.069	0.224	

Only factor loadings above 0.35 are indicated. ^a^PL: place identity items; in the original AIQ-IV questionnaire, the item belongs to PE: personal, PU: public, C: collective, R: relational identity orientation scale.

**Figure 1:**
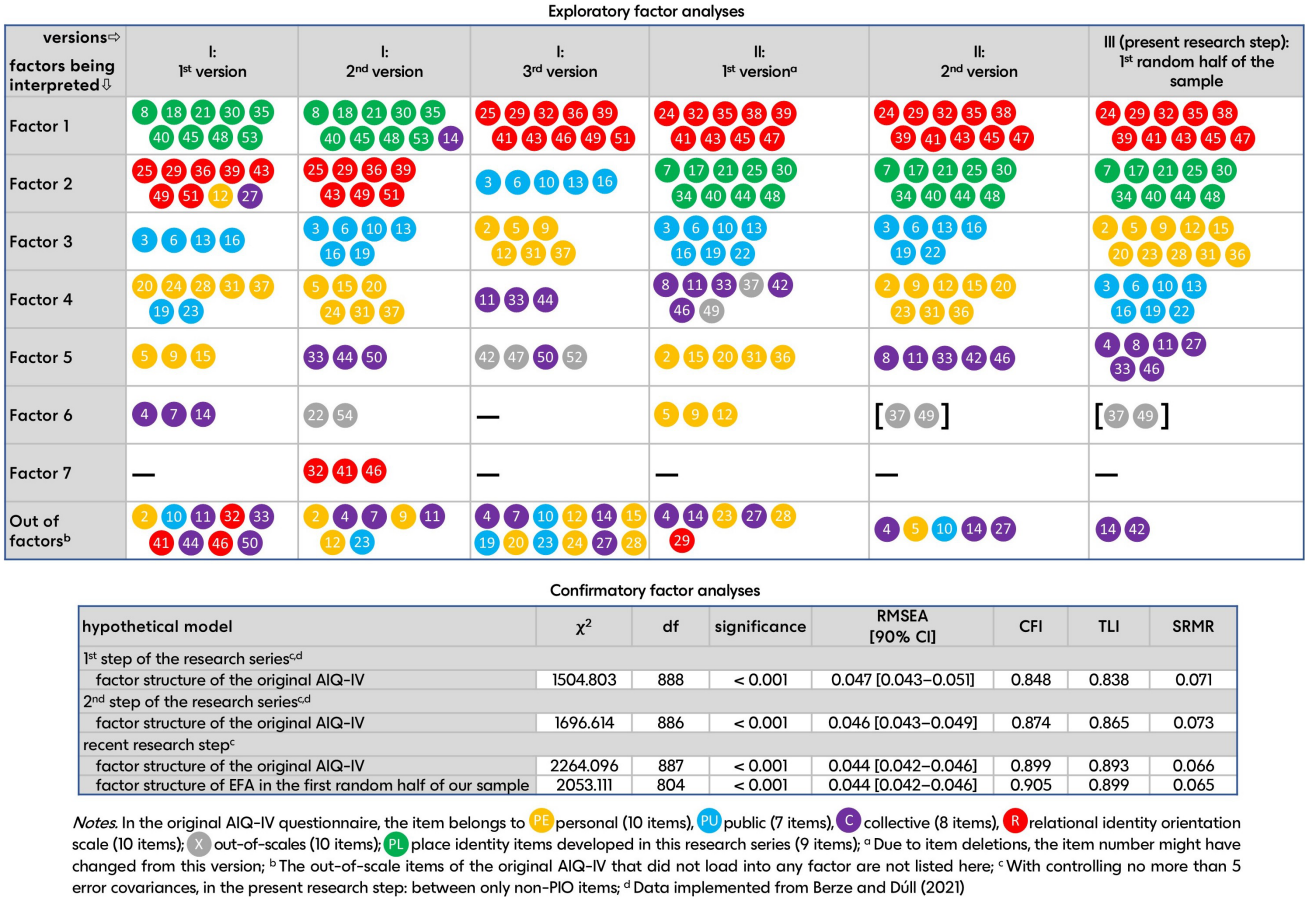
Evolution of the factor content in the three steps (I–III) of the research series according to the results of exploratory factor analyses (EFA) conducted with principal axis factoring extraction method and Promax rotation, and the fit indices of the confirmatory factor analyses (CFAs) in the steps so far of our research series.

The results of the CFA of the identity orientation items showed an adequate fit of our data of the second half of the sample to the factor structure of EFA presented above [*χ*^2^(804) = 2053,111, *p* < 0.001, RMSEA = 0.044 [90% CI: 0.042–0.046], CFit = 1.000, CFI = 0.905, TLI = 0.899, SRMR = 0.065]. The factor loadings of all items were significant (*p* < 0.001). According to the STDYX output of Mplus, the two items having loadings below 0.4 in EFA (item PU22, PU10, and C4) loaded into their factors with coefficients highly above 0.4, i.e., 0.477, 0.599, and 0.489, respectively. Error covariances between PIO items were not found that decreased fit significantly. Furthermore, the data showed the best fit to the factor structure of the original questionnaire among the versions of our research steps so far [*χ*^2^(887) = 2264.096, *p* < 0.001, RMSEA = 0.044 [90% CI: 0.042–0.046], CFit = 1.000, CFI = 0.899, TLI = 0.893, SRMR = 0.066]; however, the fit could not completely be acceptable as good regarding the values of CFI and TLI. Figure 1 shows the evolution of the factor content in the three steps of the research series and the fit indices of the CFAs in the steps so far.

According to these results, our questionnaire, which might be called hereinafter Aspects of Identity Questionnaire with Place Identity—Hungarian Version (or AIQPI-H) in this study, has five subscales: the place identity orientation, the relational, the personal, the public, and the collective identity orientation subscales, whose scores can be ranged between 9 and 45, 10 and 50, 10 and 50, 7 and 35, and 6 and 30, respectively. The values of Cronbach alpha showed great reliability of these scales (in our whole sample: 0.907, 0.914, 0.856, 0.842, and 0.785, respectively). Focusing on the place identity orientation items developed in our research series, respondents of the whole sample actually reached scores between 9 and 45 (M = 28,70, SD = 7,052) on the PIO subscale, i.e., their scores covered the entire spectrum of subscale’s reachable scores.

The PCA of PEB items (KMO = 0.816; Bartlett test: *p* < 0.001) revealed one component with an Eigenvalue greater than 1, i.e., the items were included in a single component, which explained 48.24% of the variance. Factor loadings on this component ranged from 0.78 to 0.45 and are presented in Table 2. The internal consistency of the items (Cronbach’s alpha = 0.774) was good. In the results of the item-total analysis, there is no item whose deletion would considerably increase the value of Cronbach’s alpha. The results support that a total summed score can be calculated for the responses on six PEB items to measure pro-environmental behavior. The total score could have ranged between 6 and 36.

**Table 2:** Results of the principal component analysis (PCA) of PEB items (*N* = 1,281).

Pro-Environmental Behavior items	Component 1
3. I prefer to buy environmentally friendly products.	0.783
6. I avoid the usage of single-use plastic products (bags, cutlery, cups, etc.).	0.782
2. I save water and energy.	0.766
1. Whenever I can, I collect waste separately.	0.757
5. Whenever I can, I use environment-friendly transport modes or walk instead of driving.	0.555
4. I use my things (clothes, electronics, etc.) as long as they are usable.	0.450

The descriptive statistics of the variables used in the path model analysis are reported in Table 3. The Spearman correlation matrix of the identity orientation and environmental variables is shown in Table 4.

**Table 3:** Descriptive statistics of the predictor and outcome variables used in the path analyses.

	N	%	M	SD	Min	Max
Place identity orientation	1,280		28.74	7.068	9	45
Relational identity orientation	1,281		41.00	6.503	10	50
Personal identity orientation	1,278		40.50	5.773	10	50
Public identity orientation	1,278		23.50	4.639	7	35
Collective identity orientation	1,279		18.13	4.605	6	30
Pro-environmental behavior	1,239		26.05	5.439	6	36
Connectedness with nature (INS)^a^	1,236		*4.20*	*1.337*	*1*	*7*
“1”	16	1.3				
“2”	109	8.8				
“3”	247	20.0				
“4”	370	29.9				
“5”	300	24.3				
“6”	123	10.0				
“7”	71	5.7				
Gender	1,281					
Men	430	33.6				
Women	851	66.4				
Age	1,281		29.65	13.319	18	82

^a^As more research considering and using the INS score as a continuous variable can be found in literature, although we considered the INS score as a categorical (ordinal) variable, the mean is also shown in the table in Italics.

**Table 4:** Spearman correlation matrix of the identity orientation and environmental variables (*N* = 1,281).

	(1)	(2)	(3)	(4)	(5)	(6)
Place identity orientation (1)	–					
Relational identity orientation (2)	0.347***	–				
Personal identity orientation (3)	0.231***	0.619***	–			
Public identity orientation (4)	0.274***	0.364***	0.271***	–		
Collective identity orientation (5)	0.453***	0.196***	0.108***	0.212***	–	
Pro-environmental behavior (6)	0.174***	0.188***	0.177***	0.069*	0.072*	–
Connectedness with nature (INS) (7)	0.179***	0.075**	0.056	0.009	0.100***	0.329***

*p < 0.05, **p < 0.01, ***p < 0.001.

**Figure 2:**
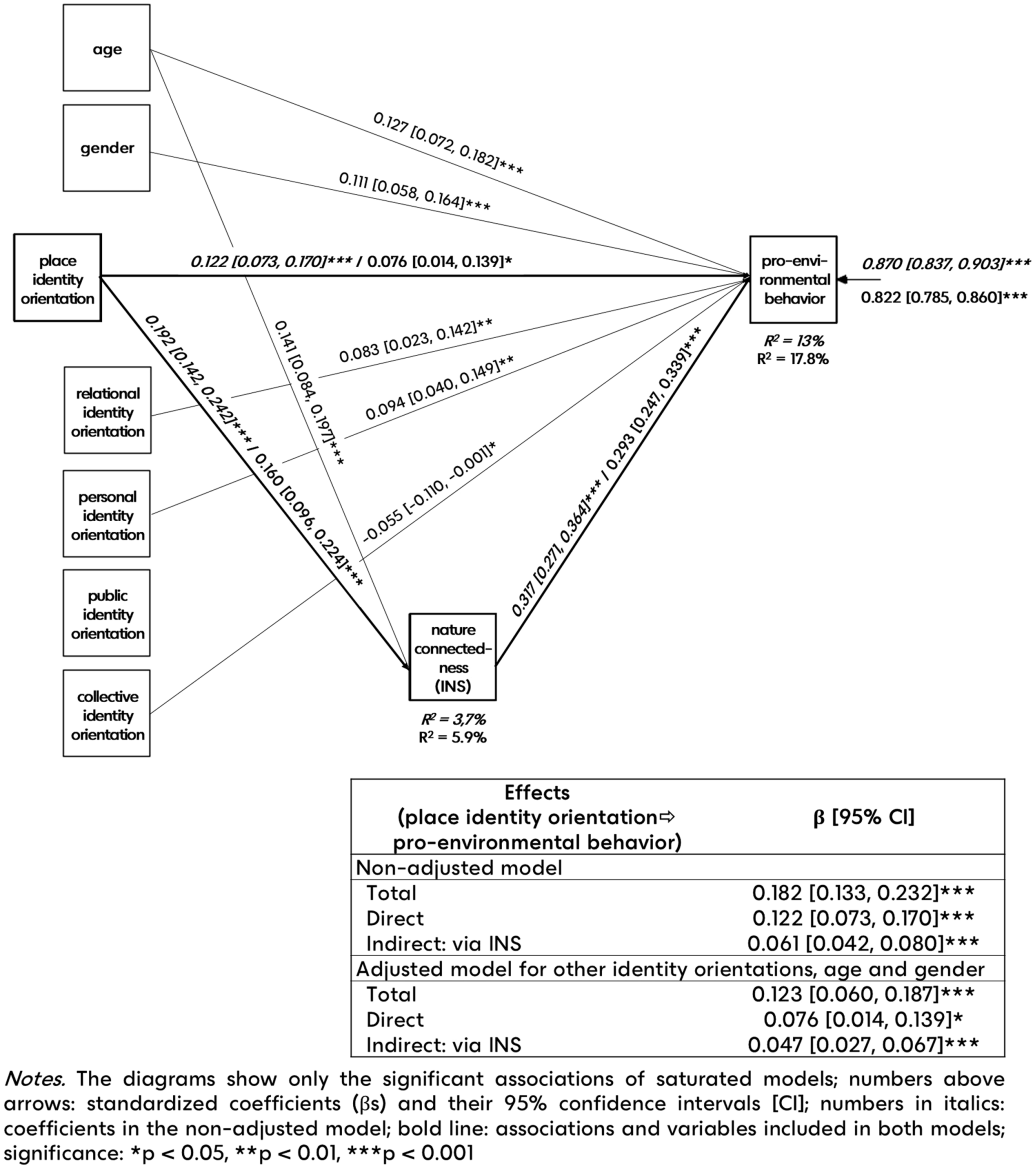
Results of the non-adjusted and adjusted path analyses (*N* = 1,281).

The non-adjusted path model (see Figure 2) showed that place identity orientation (PIO) had a weak positive association with nature connectedness (INS): respondents with higher importance of the relationship with places in their self-definition feel a higher degree of inclusion of nature in self. Furthermore, INS had a moderate positive association with PEB: respondents who feel a higher degree of inclusion of nature in their selves are more likely to behave pro-environmentally. The β of the total effect of the non-adjusted model indicated that PIO showed a weak positive association with PEB: respondents with higher importance of the relationship with places in their self-definition are more likely to behave pro-environmentally. Regarding the significant positive direct effect, partial mediation was found between place identity orientation and PEB, i.e., pro-environmentally behavior reported as more typical was associated with stronger place identity orientation not only through the higher nature connectedness (INS). The same results were found in the adjusted model, which included the other four identity orientations, age, and gender; i.e., the results above were independent of these covariates.

Since the covariates of the adjusted model were included to control them, i.e., to explore the independence of the results of the non-adjusted model, their associations are only briefly reviewed. Among the identity orientations, only place identity orientation was associated with nature connectedness. Associations were found (1) between relational identity orientation and PEB (+), (2) between personal identity orientation and PEB (+), and (3) between collective identity orientation and PEB (−); moreover, (4) between age and PEB (+), (5) between gender and PEB (women and older people are more likely to behave pro-environmentally). Finally, (6) older people showed stronger nature connectedness. Public identity orientations had no association with PEB.

## 4 Discussion

### 4.1 Interpretations of the results

Since the fit indices of the answers of the second half of the sample indicated a good fit to the factor structure that emerged in the first half of the sample, it is argued that, as a result of our research series, a questionnaire measuring place identity orientation (PIO) was developed with a factor structure considered stable. The place identity orientation scale of our AIQPI-H shows great reliability, can result in a wide spectrum of available points, and, according to the absence of considerable error covariances, includes items covering well-distinct aspects/dimensions of place identity interpretable without referring to specific places, emphasizing mainly the cognitive level of the concept. The scale thus reliably measures individual differences in tending to orient toward places when constructing self-definition. According to the results, place identity orientation seems to be separate from of the other four identity orientations. The construct of place identity orientation can be argued to suggest that people’s relationship to places is accessible on a general level. Measuring this general tendency might contribute to revealing the issues in people–environment fit in several environmental settings.

Furthermore, regarding the fit indices relative to the factor structure of the original AIQ-IV, the data of our second half of the sample showed an approximately good fit to the original questionnaire’s factor structure. This means that our questionnaire adequately measures the four identity orientations of the AIQ-IV in Hungarian samples, with one important caveat. Although, as a result of the wording modifications in our research series, three of our four identity orientation factors included all the items of the appropriate subscales of the original AIQ-IV, the items of the original collective identity orientation scale did not load into a common factor. The item focusing on the commitment to political issues (item 42) and the item concerning the places and communities where the respondent lives or was raised (item 14) were not included in the collective identity orientation factor. The former, also considering this item’s loading into the collective factor in previous versions of our questionnaire, is not astonishing regarding its dependence on a country’s prevailing political contexts and tendencies. The latter, however, can be considered as validating our new place identity orientation subscale since although this item’s wording contains the expression “place,” it emphasizes communities there and, in line with this, is not loaded into our PIO factor. In summary, the collective identity orientation subscale in our AIQPI-H questionnaire contains only 5 of the original seven adequate items. The data, on the whole, proved our expectations in H1.

The explained variance should also be discussed, whose value was not reported by Cheek et al. (2002) in the case of the original AIQ-IV. Our latest questionnaire’s items loading in one of the interpreted five factors explained 43,753% of the variance. This ratio is the highest among the questionnaire versions of our research series but remains below the expected value in social sciences. However, in such a complex domain of psychology, a value approaching 50% might be argued to be considered acceptable.

According to the results of path analyses, as expected in H2, stronger PIO, i.e., higher importance of the relationship with places in self-definition, is associated with a stronger connectedness with nature, i.e., a higher degree of inclusion of nature in self. The weakness of their association reflects the differences between the scopes of the targeted environments in the two constructs. In accordance with the occasional positive relationship, discussed in literature, of place identity with environmental behavior, and as hypothesized in H3, we found a positive validating association of PIO with PEB, independently of other identity orientations. Furthermore, as expected in H4, the positive association between PIO and PEB was achieved not only via stronger nature connectedness, i.e., higher PIO is associated with more pro-environmentally behavior not only when it meets stronger nature connectedness, but PIO also had a direct positive association with PEB. This might reflect that general PEB, although it ultimately targets protecting nature, is usually executed in the person’s everyday place-scaled environmental settings (e.g., energy saving, choosing sustainable transport, etc.) and thus requires effective, adequate, and heedful dealing with several settings of the immediate environment. Place identity can actually promote this deal. Although the direct PIO–PEB association suggests the role of the relative importance of the relationship with places in self-definition in pro-environmental behavior, further studies are required to explore the variables underlying this association.

Although the other identity orientations were included in the adjusted model as covariates to explore the independence of the results of the non-adjusted model, it is worthwhile to review their associations briefly. Regarding the associations of covariates of the model with pro-environmental behavior, several research found a relationship between self-identity and (intention to) PEB: e.g., pro-environmental self-identity and carbon offsetting identity (Whitmarsh and O'Neill, 2010: behavior), self-identification as a (household) recycler (Nigbur et al., 2010: intention and behavior; Terry et al., 1999: intention), in environmental protection (Lee, 2009: behavior), and in environmental activism (Fielding et al., 2008: intention). Similarly, our results about the positive association between pro-environmental behavior and personal identity orientation emphasize the role of personal values, thoughts, feelings, goals, and views of self in pro-environmentalism. When we think of close relationships as the platform for sharing values, thoughts, feelings, and goals (related to or independent of pro-environmentalism) with someone of mostly similar characteristics, the positive associations between environmental beliefs and relational identity orientation can be interpreted relevantly. Several research has examined the relationship of social identity with PEB and the intention to behave pro-environmentally, mainly using the concept in the interpretation of social identity theory, i.e., as group membership. When social identity was interpreted in a pro-environmental context, e.g., as group membership of environmentalists (Dono et al., 2010), positive associations with PEB were found, whereas interpreted in a more general level [as group membership in local community (Murtagh et al., 2012; Rees and Bamberg, 2014), or as membership in reference group (Terry et al., 1999)], it showed no relationship with PEB. Similarly, the public identity orientation in our research had no associations with PEB, although it does not allude to group membership (see its renaming by Cheek and Cheek (2018) cited above). Finally, the collective identity orientation items of AIQ-IV basically refer to the involuntary social group membership, i.e., family, ethnicity, religion, nation, and mother tongue, thus having a conservative tinge. The opposition of conservatives toward pro-environmentalism revealed by several studies (e.g., Ballew et al., 2019; Jylhä et al., 2016; Kukowski et al., 2023; McCright and Dunlap, 2011) might be the background to collective identity orientation’s significant, even if just at the threshold (*p* = 0.05) and very weak, negative association with PEB.

Regarding gender and age, although their association with PEB is less clear, several research confirmed higher levels of pro-environmentalism among women (e.g., Luchs and Mooradian, 2012; McCright and Xiao, 2014; Zelezny et al., 2000), and several studies explored more/stronger pro-environmental behavior among older people (e.g., Barr et al., 2001; Pinto et al., 2011; Whitmarsh and O'Neill, 2010). The positive association of nature connectedness with age is also discussed in literature (e.g., Hunt et al., 2017; Zhang et al., 2014).

### 4.2 Limitations

Among the limitations of our study, the cross-sectional design and, hence, the lack of possibility for causal inferences are worth mentioning. Since environment-relevant identities are also formed in everyday transactions with the surrounding environment, the pro-environmental actions achieved might shape and enrich them. Regarding sociodemographic characteristics, our non-representative sample is described as an overrepresentation of women and under-25s. Furthermore, the study did not include socioeconomic covariates, such as socioeconomic status, that might have a role in PEB (e.g., Eom et al., 2018).

### 4.3 Future research directions

Future research might cover the testing of the already completed English version of our questionnaire, which contains the items of AIQ-IV in their original number and wording and our PIO items translated into English in an English-speaking adult sample. In addition, it seems to be worthwhile to examine the associations of PIO with other concepts of nature connectedness, including other facets of nature–identity connection in the analysis, and with pro-environmental beliefs measured by a version of the New Ecological Paradigm (NEP) Scale (Dunlap et al., 2000). What makes the latter particularly significant is that in our other research projects justifying the multidimensionality of the Hungarian-translated version of the NEP Scale for Children (Manoli et al., 2007), one of the three subscales of the questionnaire (called Questioning of Human Intervention) showed negative associations with PEB (Berze et al., 2022) and nature relatedness (Berze et al., 2023). Based on these results, we argued that the original interpretations of this items of subscale, intended by NEP authors to be anti-environmental, have changed over the past decades as a result of the emerging emphasis on environmental protection: the human intervention is already not interpreted as clearly harmful, and the human control over their activities is inseparably connected with the control and rule over nature.

For conceptual and methodological reasons, and with the aim of being able to apply it later in projects examining people–environment fit in several place-scaled settings, we decided, as a first scanning step, to measure environment-relevant identity in the context of identity orientations at the scale and boundedness of places. However, as, based on intentional wording of some items, the participants might have thought of both built and natural environments, it cannot be stated with certainty that some of them did not also include in their answers the large-scale natural “place,” i.e., nature. At the same time, we think it might be worth considering interpreting the orientation toward the physical environment in self-definition at a more general level, i.e., level covering settings of larger and smaller scales of the environment, not only the scale and boundedness of places. The development process, prepared by our Environmental Psychology Lab, of a questionnaire measuring this “environment-relevant identity orientation” or “space identity orientation” must require several conceptual and formulating-related considerations.

Finally, the PIO-PEB partial mediation can be considered to validate our new concept. However, the relationship between PIO and “general place-scaled environmental behavior,” related to the diagnostic and problem-solving functions of place identity described by Proshansky et al. (1983), would mean a stronger validation. A tool aiming to measure it is in the process of development in our Lab.

## 5 Conclusion

As a new approach nested in the intersection between environmental and personality psychology, we argued that one of the most important concepts of environmental psychology, place identity, can also be interpreted in a personality psychological context. This new concept, place identity orientation, can be conceptualized as an individual tendency (or disposition) to orient toward the place-scaled socio-physical environments when constructing self-definition, in other words, as the relative importance of the psychological relationship with places “in general” in the person’s sense of who they are. As a result of our research series, a valid Hungarian language questionnaire measuring this new concept was developed and validated. Testing for the English version is in progress. We argue that our concept, place identity orientation, and applying the questionnaire measuring it might be useful in projects in research and practice investigating people–environment fit in several place-scaled settings. In addition, regarding the partial mediation between PIO and PEB found in the present research, our concept might also serve as a new perspective in studying and motivating pro-environmental behavior.

## Data Availability

The raw data supporting the conclusions of this article will be made available by the authors, without undue reservation.
